# Bites by the Monocled Cobra, *Naja kaouthia*, in Chittagong Division, Bangladesh: Epidemiology, Clinical Features of Envenoming and Management of 70 Identified Cases

**DOI:** 10.4269/ajtmh.16-0842

**Published:** 2017-04-05

**Authors:** M. A. Faiz, M. F. Ahsan, A. Ghose, M. R. Rahman, R. Amin, M. Hossain, M. N. U. Tareq, M. A. Jalil, U. Kuch, R. D. G. Theakston, D. A. Warrell, J. B. Harris

**Affiliations:** 1 Dev Care Foundation, Dhaka, Bangladesh; 2 Department of Zoology, University of Chittagong, Chittagong, Bangladesh; 3 Chittagong Medical College and Hospital, Chittagong, Bangladesh; 4 Department of Medicine, Shaheed Suhrawardy Medical College, Dhaka, Bangladesh; 5 Dhaka Medical College and Hospital, Dhaka, Bangladesh; 6 Chittagong General Hospital, Chittagong, Bangladesh; 7 Department of Statistics, University of Dhaka, Dhaka, Bangladesh; 8 Institute of Occupational Medicine, Social Medicine and Environmental Medicine, Goethe University, Frankfurt am Main, Germany; 9 Alistair Reid Venom Research Unit, Liverpool School of Tropical Medicine, Liverpool, United Kingdom; 10 Nuffield Department of Clinical Medicine, John Radcliffe Hospital, University of Oxford, Oxford, United Kingdom; 11 Medical Toxicology Centre and Institute of Neurosciences, Newcastle University, Newcastle upon Tyne, United Kingdom

## Abstract

We describe 70 cases of monocled cobra (*Naja kaouthia*) bite admitted to Chittagong Medical College Hospital, Bangladesh. The biting snakes were identified by examining the dead snake and/or detecting *N. kaouthia* venom antigens in patients' serum. Bites were most common in the early morning and evening during the monsoon (May–July). Ligatures were routinely applied to the bitten limb before admission. Thirty-seven patients consulted traditional healers, most of whom made incisions around the bite site. Fifty-eight patients experienced severe neurotoxicity and most suffered swelling and pain of the bitten limb. The use of an Indian polyvalent antivenom in patients exhibiting severe neurotoxicity resulted in clinical improvement but most patients experienced moderate-to-severe adverse reactions. Antivenom did not influence local blistering and necrosis appearing in 19 patients; 12 required debridement. Edrophonium significantly improved the ability of patients to open the eyes, endurance of upward gaze, and peak expiratory flow rate suggesting that a longer-acting anticholinesterase drug (neostigmine) could be recommended for first aid. The study suggested that regionally appropriate antivenom should be raised against the venoms of the major envenoming species of Bangladesh and highlighted the need to improve the training of staff of local medical centers and to invest in the basic health infrastructure in rural communities.

## Introduction

**Figure 1.**
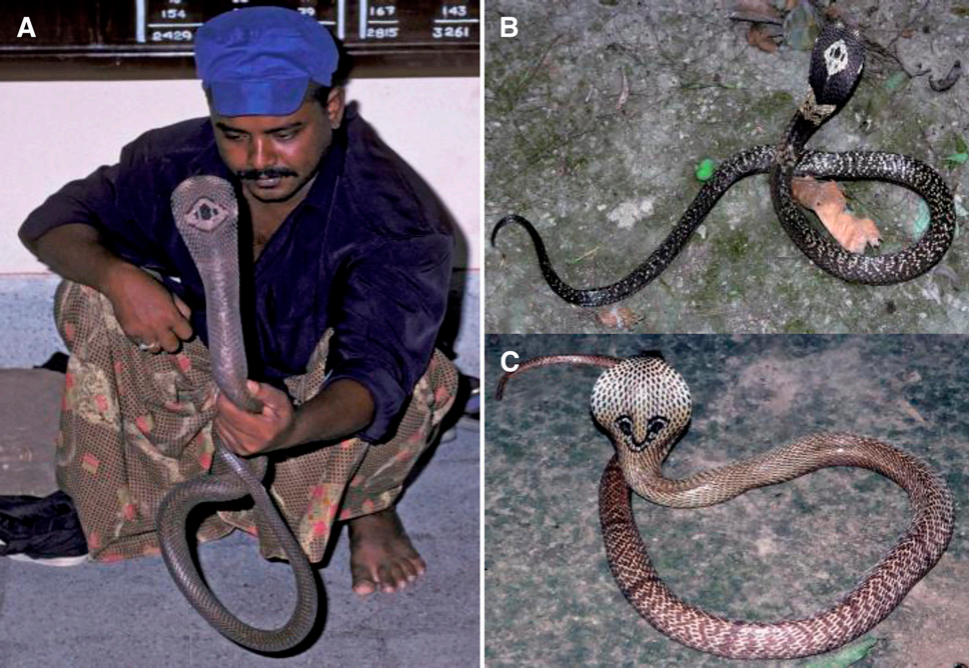
(**A**) Monocled cobra (*Naja kaouthia)* from southeastern Bangladesh with snake handler. (**B**) *Naja kaouthia* from Kolkata, West Bengal, India. Note the variation in the pattern of the monocular hood markings between A and B. (**C**) Spectacled cobra (*Naja naja)* from Kolkata, West Bengal, India, showing the distinctive “pair of “spectacles”.

The venomous snake fauna of Bangladesh remains poorly characterized, but clinical reports from the regions of Chittagong, Cox's Bazaar, Khulna, Rajshahi, and Mymensingh have described neurotoxic envenoming, a life-threatening medical emergency that requires treatment with specific antivenom and assisted ventilation in cases of respiratory paralysis.^1^–^8^ In Asia and Africa, envenoming by some cobra species is associated with substantial local soft tissue damage requiring surgical debridement often leading to prolonged hospitalization, scarring, malignant transformation, and potentially permanent disability.^9^–^14^ The present study of bites by monocled (monocellate) cobras, *Naja kaouthia* (Figure 1A and B

), forms part of a large prospective study of snakebite in Chittagong Division, Bangladesh. This division located in southeastern Bangladesh, covers an area of 33,771 km^2^ with a population of approximately 28 million. The study was based at Chittagong Medical College Hospital (CMCH), the major tertiary care referral hospital for the Division and was carried out between May 1999 and October 2002. Most patients were received from Chittagong, Cox's Bazaar, and the three Chittagong Hill Tracts districts. We hoped to define, more precisely, the clinical phenotype of envenoming, assess antivenom use and provide a rational basis for defining national strategies required to improve medical care. Eight hundred and eighty-four patients with a credible history and signs and symptoms of snakebite envenoming were admitted. Of the 884 patients, 70 were identified as victims of bites by *N. kaouthia* by examining the snake responsible, if brought to the clinic, and/or by the detection of the specific venom antigens in the patient's serum. Here, we describe the epidemiology, prehospital treatment, clinical assessment, management, and outcomes of these 70 patients.

## Materials and Methods

### Case history.

A full case history was completed for each patient, including personal and epidemiological data and details of prehospital treatment, using a standard pro forma. Physical signs consistent with snakebite (fang marks, oozing of blood from putative fang punctures, localized pain, and swelling) were recorded. Evidence of bleeding from old wounds as well as spontaneous bleeding from the gums and external orifices, and bloody sputum, feces, or urine was noted. A neurological examination was made to detect signs of neurotoxicity (such as bilateral ptosis, external ophthalmoplegia, dysphagia, dysphonia, reduced grip strength, weak neck muscles, and “broken neck sign”) and/or myotoxicity (such as muscle pain or tenderness and elevated serum creatine kinase). The ability to open and close the mouth, to protrude the tongue, and to cough was assessed, as well as chest movements, respiratory difficulty, pooling of oral secretions in the oropharynx, and breathlessness. Blistering and necrosis near the site of the bite were recorded. Blood pressure and heart rate were routinely measured. Venous blood samples were collected for hematological measurements and coagulability testing using the 20-minute whole blood clotting test (20WBCT).^15^,^16^

### Antivenom administration.

Haffkine polyvalent antivenom (raised against the venoms of *Naja naja*, *Bungarus caeruleus*, *Daboia russelii*, and *Echis carinatus* sourced mainly from Mamallapuram, Tamil Nadu, India) was administered to all patients exhibiting signs of advanced systemic neurotoxicity (e.g., bilateral complete ptosis, external ophthalmoplegia, dysphagia, dysphonia, weak grip strength, weak neck muscles, “broken neck” sign, or respiratory difficulty). Antivenom was not given to patients with minimal signs of neurotoxicity (e.g., partial ptosis with no other signs of systemic neurotoxicity) or to those with local signs of envenoming (e.g., blistering and local necrosis) in the absence of systemic neurotoxicity. Antivenom use conformed to Bangladeshi national guidelines: 100 mg of immunoglobulin in 100 mL vehicle was made up to 200 mL in 0.9% w/v NaCl as directed by the manufacturer and administered via intravenous infusion over a period of 1 hour. In the absence of any reversal of paralysis after 2 hours, the same dose was repeated. Infusion was suspended if a patient developed adverse responses (e.g., urticaria, anaphylaxis, severe pyrogenic side effects) before the infusion was completed. In that event, adrenaline (0.25 mg i.m.), (0.2 mg/kg i.v.), and hydrocortisone (2 mg/kg i.v.) were administered and the infusion of antivenom was resumed.

### Ventilatory support.

Ventilatory support was provided to those patients exhibiting cyanosis, sighing respiration, or other signs of ineffective ventilation. Support was withdrawn as soon as adequate spontaneous respiration was reestablished and the patient resisted enforced ventilation.

### Detection of venom antigen in the serum of patients.

A micro enzyme-linked immunosorbent assay technique (micro-ELISA) was used to detect venom antigens in the serum of the bitten patients.^17^,^18^ Microtiter assay plates were coated with rabbit anti-*N. kaouthia* IgG as the antigen capture system. This enabled the formal identification of the biting snake and also the calculation of venom antigen concentration in the serum from a standard venom concentrations curve ranging from 0.1 to 500 ng/mL in phosphate-buffered saline (PBS; pH 7.2) containing 1% v/v normal rabbit serum. Specificity was confirmed using the venoms of four major biting species in Bangladesh (*B. caeruleus*, *N. kaouthia*, *Trimeresurus albolabris*). The lower limit of detection was 0.5 ng/mL. Venom antigen data were subsequently reconciled with previously collected clinical records by J. B. Harris. Urine was examined for the presence of albumin and/or erythrocytes.

### Effect of *N. kaouthia* venom on blood coagulation.

A lyophilized, pooled sample of the venom of *N. kaouthia* from a fully documented venom collection held by the Venom Research Unit, Liverpool School of Tropical Medicine, was used to determine the effects of the venom on activated partial thromboplastin times (APTT) and thromboelastography (TEG) under controlled laboratory conditions. A pooled sample of the venom of *Pseudechis papuanus* from Papua New Guinea was used as a positive control. Venom samples were stored at −80°C until reconstituted for use in PBS (pH7.2) and aliquots of 50 μL were stored at −80°C. When required, the venom was thawed at 37°C then placed on ice for the duration of the experiment. Serial dilutions of venom were made in PBS to provide venom concentrations ranging between 35 and 4,436 ng/mL.

Control blood samples for measuring the effects of the venoms on APTT and TEG were obtained from 10 healthy volunteers (five females, age range 22–52 years; five males, age range 23–50 years) and collected into 0.105 M sodium citrate. Plasma, obtained by centrifugation of the citrated blood (1,780 × *g*) for 10 minutes, was pooled before use.

The effects of snake venom on human blood coagulation were evaluated using the Amelung KC4 (Trinity Biotech PLC, Wicklow, Ireland). Briefly, 100 μL of citrated human plasma was introduced to the KC4 cup, followed by 100 μL of IL Hemosil APTT reagent (Instrumentation Laboratories Company, Bedford, MA) and 10 μL of appropriately diluted snake venom and the mixture incubated for 220 seconds. Clotting was triggered by the addition of 100 μL 0.02 mol/L CaCl_2_ (Instrumentation Laboratories Company, Bedford, MA) and the clotting time recorded.

TEG analysis was performed using a TEG 5000 Homeostasis analyzer (Hemonetics Corporation, Braintree, MA). Blood was collected from healthy volunteers into 0.105 M sodium citrate tubes, mixed, and then allowed to stand at room temperature for 30 minutes before testing. All TEG tests were performed within 2 hours of venepuncture. To test the effects of venoms on the TEG, 10 μL of appropriately diluted snake venom was added to the incubation cup together with 20 μL of 0.2 M CaCl_2_. One milliliter of citrated blood was added to a vial containing kaolin (Haemonetics Corporation). The kaolin/blood mixture (330 μL) was added to the incubation cup and the resulting TEG trace was recorded for 45–60 minutes. Each experiment was completed three times on different samples of plasma/blood as appropriate.

### The Tensilon^®^ test.

A Tensilon^®^ (edrophonium) test was used in a separate group of 18 randomly recruited cobra bite patients exhibiting clinical signs of neurotoxicity. A randomized, double-blind, crossover design was used, each patient serving as his or her own control. Whether edrophonium or placebo (the corresponding volume of NaCl 0.9% w/v) was to be administered first was determined by lottery; neither observer nor patient knew which was being given. Atropine sulfate (0.6 mg i.v.) was given to all patients beforehand. Either edrophonium (10 mg i.v.) or placebo was given immediately afterward by intravenous infusion over 2 minutes. Thirty minutes later patients received, in reverse order, either edrophonium or placebo as required. The duration for which a patient could retract the eye lids, the area (%) of the iris exposed when a maximal effort was being made to open the eyes, the ability to open the mouth, peak expiratory flow rate (PEFR), and the maximum pressure generated by the patient when trying to blow into a manometer were measured every 5 minutes for 20 minutes after the administration of edrophonium or placebo. One patient had received antivenom before the initiation of the test, two were given antivenom during the test, and 14 were given antivenom after the completion of the test. A single patient for whom antivenom was unavailable was maintained on neostigmine, 50 mcg/kg body weight subcutaneously every 4 hours following the completion of a positive edrophonium test, until the neurotoxic signs disappeared.

### Ethical considerations.

All patients, or a close relative where necessary, gave written informed consent for the inclusion, in public, academic, and professional presentations and publications, of personal, circumstantial, clinical, and laboratory information, including photographic and radiological images relating to medical advice, diagnosis, and treatment received by the patient at CMCH. Standardized data forms containing all personal data, including details of treatment, were securely stored at CMCH. These forms were exclusively available to clinical staff directly involved in the care of the patients. Anonymized data associated only with a patient ID number were stored electronically in an SPSS database that was available to all involved with the research project.

### Statistical analysis.

Numerical data are given as mean ± standard error or as median and range as appropriate. Data were analyzed using SPSS version 21 software (SPSS Inc, Chicago, IL). Differences between different data sets were calculated using Students' *t* test or the Mann–Whitney test as appropriate. *P* < 0.05 was considered indicative of statistical significance. Tests for correlations between two independent sets of data were made using Pearson's test.

## Results

### Case studies.

Case 1: A 7-year-old girl from Azimpur village, Patiya Upazilla (subdistrict), was bitten on her left ankle at home at 10:00 pm by a “blackish/yellowish snake” approximately 68 cm long. The snake was caught, killed, and eventually brought to CMCH where it was formally identified as “zhawra,” the local name for a monocled cobra, *N. kaouthia*, based on the distinctive single eye marking on the dorsal surface of the hood. The patient reported immediate pain and there was localized bleeding at the bite site. She was taken without delay to a traditional healer, locally known as an ohza, ozha, ojha, or bede, who applied three ligatures proximal to the bite site, two on the calf and one on the thigh. The bite site was incised and she was given a herbal infusion to drink. She was admitted to CMCH between 3 and 4 hours after the bite. Ligatures were released and amoxicillin (10 mg/kg i.v.) and neostigmine (50 μg/kg s.c.) were administered. The pulse rate was 104 beats/minute and blood pressure 105/80 mmHg. Two fang marks, 10 mm apart, were identified. The patient felt faint and generally weak and had difficulty in swallowing and speaking. Five hours after the bite, neurological signs predominated with complete bilateral posies, external ophthalmoplegia, an inability to open or close the mouth or protrude the tongue, a “broken neck” sign, a weak grip, and depressed reflexes. Peak flow was 100 L/minute. Total white cells (15 × 10^9^/L cf. reference range 4–11 × 10^9^/L) and polymorphs (82% cf. reference range 20–40%) were elevated, but platelet counts and hemoglobin concentrations were within normal limits.

Antivenom (Haffkine polyvalent 100 mg in 200 mL) was infused intravenously. Neurotoxic signs began to diminish after 35 minutes. Adverse reactions to antivenom (urticaria, cough, wheezing, and angio-edema of the lips and tongue) were treated with adrenaline (0.25 mg i.m.), chlorphenamine (0.2 mg/kg i.v.), and hydrocortisone (2 mg/kg i.v.). Albumin (concentration not recorded) was detected in the urine but there were no erythrocytes or casts. Electrocardiogram was normal. By 2 days after the bite, all neurotoxic signs had resolved but a small area (∼3 cm diameter) of blistered, necrotic tissue appeared. The blister was aspirated and necrotic tissue debrided. The patient was discharged 8 days after admission. The circulating concentration of *N. kaouthia* venom antigen was 3.9 ng/mL.

Case 2: An 18-year-old agriculture worker from Raojan was walking along a drainage ditch at 7:00 pm. He was bitten on the dorsal aspect of the right foot by a “blackish snake, approximately 120 cm long and 3 cm in diameter,” which he identified as “zhawra.” Three ligatures were applied immediately, two to the bitten leg and one to the thigh. The bite site was not incised and no herbal remedies were used. He was not seen by a traditional healer. He did not vomit or feel faint but developed weakness and drooping eyelids 1 hour after the bite, and had difficulty in speaking and swallowing after 90 minutes. Within 2 hours, he developed neck weakness and passed scanty, highly colored urine. At 2.5 hours, he complained of breathlessness. He was admitted to CMCH 3 hours after the bite. Pulse rate was 120 beats/minute and blood pressure 110/70 mmHg. There were two fang puncture marks, 15 mm apart, to the dorsum of the right foot. Bilateral ptosis was complete and was accompanied by external ophthalmoplegia, the “broken neck” sign, weak grip and depressed tendon reflexes, an inability to open or close the mouth or protrude the tongue, pooled saliva, and an absent gag reflex. Peak flow could not be recorded and assisted ventilation was started within 1 hour of admission. He was then treated with cephradine (500 mg/kg orally), metronidazole (7.5 mg/kg orally), neostigmine (50 mcg/kg s.c.), and atropine (0.6 mg i.v.). An infusion of antivenom (Haffkine polyvalent, 100 mg in 200 mL) was given in two doses. The first dose, given at 4–5 hours postadmission, was followed within 30 minutes by an anaphylactic reaction that was managed with adrenaline (0.25 mg i.m.), chlorphenamine (0.2 mg/kg i.v.), and hydrocortisone (2 mg/kg i.v.). There was no positive response to the antivenom and the dose was repeated at 7 hours after further treatment with adrenaline. The patient remained on continuous assisted ventilation but was unresponsive until his death 7 days after admission. Immediately before death, oxygen saturation fell to 21% and he had an episode of *status epilepticus*. The cause of death was recorded as hypoxic encephalopathy.

Laboratory data confirmed elevated total white cell counts (16 × 10^9^/L cf. reference range 4–11 × 10^9^/L and polymorphs (85% cf. reference range 20–40%). Platelet counts were within normal limits. Hemoglobin concentration was below the lower level of the reference range (13 g/dL cf. reference range 14–18 g/dL). Serum creatine kinase was elevated (1,076 units/L cf. 150–200 units/L in normal subjects). The circulating concentration of venom antigen was 244.6 ng/mL.

### Epidemiology.

The average age of the patients was 29 years (median = 26 years, range 3.5–85 years) and the male/female ratio was 1.9:1. Thirty-six percent of patients were students, 26% were housewives/domestic workers, and 20% were engaged in agriculture. No bites were recorded during the cooler months of December, January, and February. Thereafter, bites became more common with rising temperatures and humidity to peak during the monsoon period of May–July. All patients were bitten on the limbs, typically below the knee or elbow. Bites were most common between 5:00 am and 12:00 pm or 3:00 pm and 11:00 pm and peak times were between 6:00 am and 10:00 am and between 6:00 pm and 7:00 pm. Only 12% of patients were asleep at the time of the bite.

### Prehospital treatment.

All 70 patients had ligatures (mean = 3, range 1–6) applied proximal to the bite site. Thirty-seven of the patients had visited a traditional healer before admission to CMCH. The typical consultation lasted between 1 and 2 hours, but in four cases lasted for > 10 hours. All patients visiting a traditional healer were incised at the site of the bite and 25 of them had been given a herbal infusion of unknown composition to drink. Mud and herbs were occasionally applied to the bite site but details of these practices were not formally collected. In only one case was the bitten limb immobilized and a pressure bandage was applied.^19^

### Clinical assessment and management.

Patients were admitted to CMCH between 1 and 27 hours (median 7 hours) after the biting incident and ligatures were slowly released. Tetanus toxoid and amoxicillin were given to 63 patients and neostigmine to 57 of them. Visible fang punctures/scratches could be identified in 32 of the 70 patients admitted. There was slight bleeding at the putative bite site in only five of them. Neither persistent bleeding from the bite site nor bleeding from any remote site was seen in any patient. Blood pressure was unremarkable in all patients. Swelling, localized to the bitten limb was seen in 53 patients, and pain at the bite site, reported by 42 patients, was transient and was treated with paracetamol, diclofenac, or ibuprofen as required. Lymph node tenderness and swelling was seen in 17 patients.

**Figure 2.**
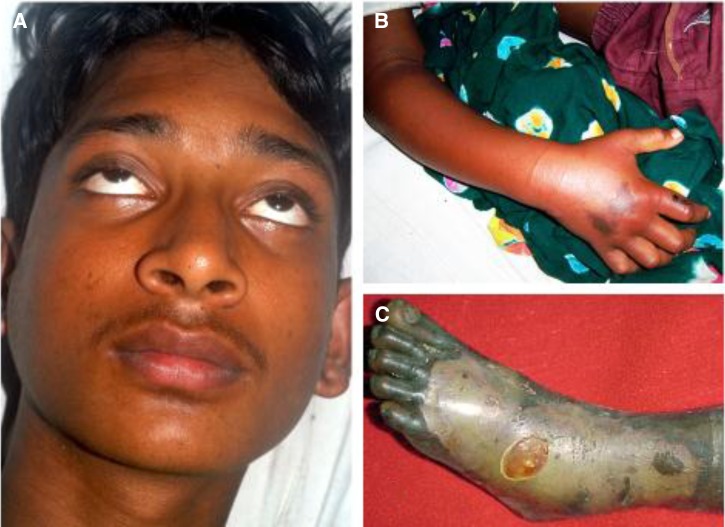
(**A**) Bilateral ptosis, typically the first sign of neurotoxicity following snakebite envenoming, was seen in 58/70 patients bitten by *Naja kaouthia* in southeastern Bangladesh. (**B**) Swelling extending above the elbow and early demarcated hyperpigmentation at the site of the bite over the proximal phalanx of the right index finger and over the dorsum of the hand. (**C**) Skin necrosis was seen in 19/70 patients in this series. Note advanced superficial necrosis and de-roofed blister following a bite on the dorsum of the foot.

One patient, who had been bitten on the foot, brought the offending snake to the clinic. It was positively identified as *N. kaouthia*. The patient developed no neurotoxicity or local blistering and necrosis and she was discharged 48 hours after the bite. Subsequent analysis confirmed the absence of venom antigen in her serum. It was concluded that she had received a bite without the injection of venom. The other 69 patients were all envenomed. Nonspecific signs/symptoms included vomiting, seen on admission in 43 patients and fainting experienced by 10 patients. Neurotoxic signs present on admission included bilateral ptosis (58 patients Figure 2A

), difficulty in speaking (52), generalized weakness (49), weak neck muscles (44), difficulty in swallowing (41), blurred vision (29), breathlessness (23), and cyanosis in three (Table 1). No patient presented with or developed generalized muscle tenderness or pain. Swelling of the bitten limb (Figure 2B) was seen in 53 patients. Localized blistering and necrosis of the skin at or close to the bite site (Figure 2C) appeared during day 1 in four patients, thereafter increasing to six by day 2, nine by day 3 to peak at 19 by day 5. In three patients, there was local blistering with no signs of systemic neurotoxicity, and in one patient the blister first appeared 5 days after admission. Surgical debridement of necrotic skin was required in 12 patients.

Antivenom was used in all 58 patients exhibiting signs of advanced neurotoxicity (e.g., bilateral ptosis, external ophthalmoplegia, “broken neck” sign, cranial neuropathy). One patient received 50 mg of antivenom and one patient received 200 mg in two divided doses. All others received a single dose of 100 mg as described in Materials and Methods section. There was clinical improvement in 51 patients, with complete resolution of ptosis, difficulties with speech and swallowing, and general neck weakness within 1 hour in some cases but on average by 5–6 hours after the initiation of treatment. In 4 cases, there was no further deterioration but neurotoxic signs continued for up to 30 hours and breathlessness for up to 50 hours. There was a continuing deterioration in the condition of three patients, and two of those three patients succumbed, one to hypoxic encephalopathy (see Case 2 above) and one to cardiorespiratory failure; the latter patient had a previous history of cardiovascular problems. It is significant to note that blistering and localized skin necrosis appeared in 16 patients already treated with antivenom. 

Eleven patients required assisted ventilation: four were ventilated for 2 hours or less, five for up to 24 hours, one for 36 hours, and one (see Case 2 above) for 7 days.

### Laboratory tests.

Hematological data are summarized in Table 2. Total white cell counts were elevated in > 80% of patients and the proportion of polymorphs was elevated in > 98% of patients. Hemoglobin concentrations were low in the majority of both male and female patients. All other hematological data (including basophile, eosinophil, and platelet counts) were similar to the accepted normal reference range for Bangladesh (CSCR), Chittagong, Bangladesh: Total leukocyte count: 4.0–11 × 10^9^/L, differential leukocyte counts: neutrophils 45–70%, lymphocytes 20–40%, monocytes 2–5%, eosinophils 1–6%, basophils 0–1%, platelets 150–400 × 10^9^/L, cyanmethemoglobin (hemoglobin) ♂ 14.0–18.0 g/dL, ♀ 12.0–16.0 g/dL). The presence of albumin in the urine was detected in 46% of patients and erythrocytes were present in 22%. There were no other signs of renal complications.

The 20WBCT was negative (i.e., blood clotted within 20 minutes in a glass vessel at ambient temperature) in all but two patients.

**Figure 3.**
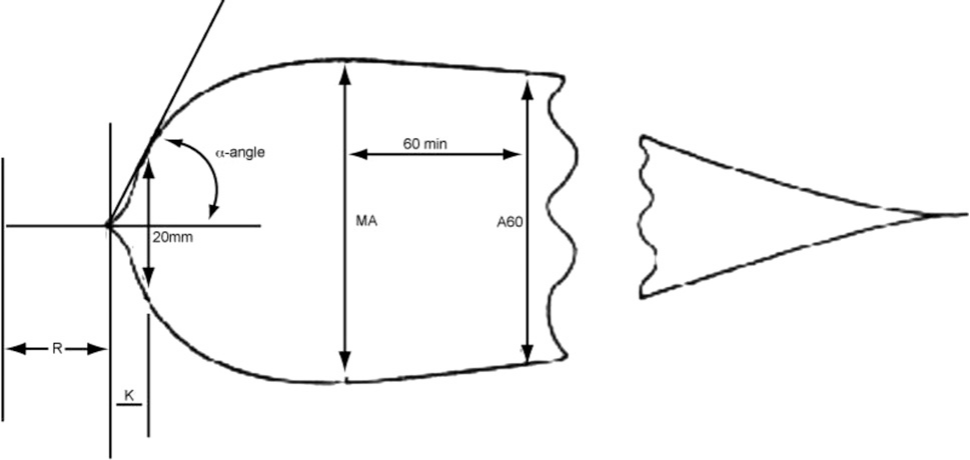
Stylized thromboelastography trace illustrating information available. R = latent period preceding activation of clot formation. K = α angle. Rate of clot formation is measured 20 minutes after the initiation of clotting. MA = clot strength.

APPT was not increased by concentrations of *N. kaouthia* venom up to 4,436 ng/mL (laboratory normal reference range 30–45 seconds; in the presence of 4,436 ng/mL venom: 34.5 ± 0.55 seconds, *N* = 10). Similarly, there were no significant changes in any of the parameters comprising TEG except for an insignificant fall in the α angle (signifying a small reduction in the rate of clot formation) at venom concentrations between 277 and 555 ng/mL. *Pseudechis papuanus* venom served as a positive control; APTT increased rapidly with increasing concentrations of venom from 3.8 ± 0.21 seconds (*N* = 10) at a concentration of 35 ng/mL to 64.6 ± 2.7 seconds at 277 ng/mL, and 241.7 ± 11.6 seconds at 4,436 ng/mL. *Pseudechis papuanus* venom also caused a slowing of the initiation of the clotting process R, and a reduction in the kinetics of clotting and clot formation (K and α angle). There was no change in the ultimate strength of the clot, MA, or the rate of breakdown of the clot that formed, G (see Figure 3

).

### Serum venom antigenemia levels.

**Figure 4.**
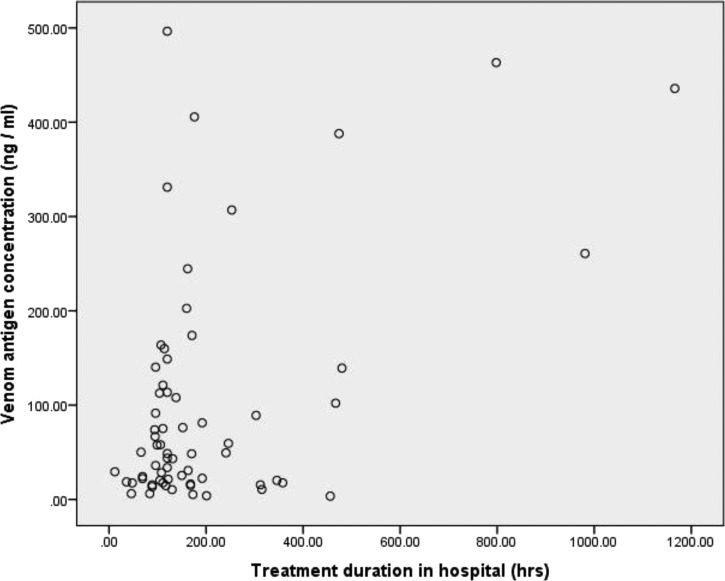
Relationship between venom antigen concentration in the patients' sera and treatment duration in hospital of 70 patients bitten by *Naja kaouthia* in southeastern Bangladesh. The Pearson correlation coefficient was 0.05 and the relationship was statistically significant (*P* < 0.05).

Venom antigenemia was calculated in 65 patients. One patient with a proven bite but no clinical signs of envenoming had no detectable venom antigenemia. In the other 64 patients, venom antigenemia ranged between 3.5 and 496.5 ng/mL (mean = 99.3, mode 49 ng/mL). There was no correlation between venom antigenemia and either the age or gender of the patient. There was a clear relationship between venom antigenemia and the clinical severity of envenoming. Thus, in five patients with only incomplete ptosis but without any respiratory difficulty, no difficulty with speech or swallowing, and no neuromuscular weakness, and for whom antivenom was deemed unnecessary, venom antigenemia ranged between 3.5 and 24.3 ng/mL (mean 13.4 ± 3.6). In those with severe neurotoxicity but no local necrosis, venom antigenemia was significantly higher (mean 103.2 ± 16.6; *N* = 39; *P* < 0.05). Those with both severe neurotoxicity and local tissue necrosis had a similarly high venom antigenemia (mean 119.2 ± 35.2; *N* = 20). The two patients who died as a result of envenoming had venom antigenemias of 244.6 and 260.8 ng/mL, respectively. There was a positive correlation (*P* < 0.05) between venom antigenemia and the duration of hospitalization (Figure 4

), but no relationship between venom antigenemia and the appearance of localized blistering and necrosis. Adverse reactions to antivenom were experienced by 68% of patients treated with antivenom. In all but one patient, in whom the antivenom use was abandoned due the severity of the adverse response, adverse reactions were successfully managed with adrenaline (0.25 mg i.m.), chlorphenamine (0.2 mg/kg i.v.), and hydrocortisone (2 mg/kg i.v.).

### The Tensilon^®^ (edrophonium) test.

The test was used in a separate group of 18 patients with neurotoxic envenoming following monocled cobra bite. The ability to open the eyes (as reflected in the percentage of iris uncovered) in all 18 patients was significantly improved after the administration of edrophonium. The improvement was first seen 5 minutes after administration when the percentage of iris exposed increased to 49% ± 6.5%, compared with 33% ± 5.4% after placebo. The improvement lasted until 15 minutes after edrophonium, when the area of iris exposed was 45% ± 7.0%, compared with 31% ± 5.6% after placebo (*P* < 0.001). The improvement of the function of the elevator palpebrae superioris muscle was also reflected in the improvement of endurance of upward gaze, over the same time period. Edrophonium also produced a statistically significant improvement in PEFR, the ability to generate pressure while blowing into the disconnected tube of a blood-pressure manometer, and the ability to open the mouth 5 minutes after administration. The data are summarized in Table 3.

### Discharge from hospital.

Patients were discharged from CMCH when attending clinicians considered them to be sufficiently recovered from their experience and were clinically stable.

## Discussion

A nation-wide community-based survey of snakebite in rural Bangladesh estimated that 589,919 individuals were bitten each year and that 6,041 of those bites were fatal.^20^ Most fatal snake-bite cases in Bangladesh bites are considered to be attributable to neurotoxic envenoming by cobras (*Naja* spp.) and kraits (*Bungarus* spp.). Differentiation between cobra and krait bites on admission to a clinical facility is important because they require different strategies for successful treatment.^8^ The determination of the biting species is obviously straightforward if the snake responsible is brought to the clinic with the patient. Usually, however, the physician will have to consider the clinical syndrome and the circumstantial evidence of the biting incident before making a decision on the most appropriate treatment for the patient.^8^,^21^

The present study of 70 patients bitten by monocled cobras (*N. kaouthia*; Figure 1A and B) is the largest and most detailed study of envenoming by this species published so far. The identity of the biting snake was established in every case, either by examining the dead snake or by the retrospective detection of venom antigens of *N. kaouthia* in the patient's serum using ELISA.^22^–^24^ The only other species of cobra known to be present elsewhere in Bangladesh is the spectacled cobra, *N. naja* (Figure 1C). The venom of this species could theoretically cross-react in the ELISA test. However, no specimen of *N. naja* could be located in any natural history or other zoological unit in southeast Bangladesh and no specimens had been obtained from the catchment area of CMCH during decades of clinical and field research. It is not recognized by local expert zoologists, or snake collectors as being a part of the local fauna.

Bites by the monocled cobra were least common during the cool and dry months of November–January, becoming progressively more common as temperature and humidity rose to peak during the monsoon season of May–July. The monocled cobra is crepuscular and biting incidents were particularly common during the early morning and evening and rare between 11:00 am and 3:00 pm and during the night. Tight ligatures were routinely applied to the bitten limb by traditional healers or by colleagues/family members of the patients. Many patients had multiple incisions inflicted around the bite-site with nonsterile knives and pieces of glass. In some cases stones, herbs and soil had been applied to the bite site. All these treatments pose a risk of secondary infection and there is a lesser but real risk of infection by the snake's oral bacteria.^25^,^26^ For these reasons, all snake-bite patients admitted to CMCH were routinely treated with tetanus toxoid and antibiotics.

Severe neuromuscular weakness associated with envenoming was typically reversed, within 5–6 hours, by treatment with the only available antivenom (Indian Haffkine polyvalent antivenom). The complete restoration of spontaneous respiration in those requiring assisted ventilation was slower but in most cases was achieved within 24 hours. The administration of antivenom did not prevent the later development of local blistering and soft tissue necrosis. The majority of patients receiving the Haffkine^®^ antivenom experienced undesirable adverse responses including, urticaria, pyrogenesis and, in one case, anaphylaxis that resulted in the cessation of antivenom administration.^27^ The Indian Haffkine^®^ polyvalent antivenom used in Bangladesh is not prepared using the venoms of local, medically important envenoming species in Bangladesh and it is unlikely that it is effective in neutralizing the effects of the necrotizing factors present in the venom of *N. kaouthia.* It is clear that regionally appropriate antivenoms for use in Bangladesh should be raised against the venoms of the major local envenoming species.

Several studies of patients bitten by either viperid or elapid snakes have demonstrated a correlation between circulating venom antigen concentration at the time of admission to hospital and the severity of the clinical syndrome.^23^,^24^,^28^–^33^ However, a study of bites by the Australian red-bellied black snake, *Pseudechis porphyriacus*, demonstrated no correlation between peak venom antigen concentration and clinical severity of envenoming.^34^ We identified several patients bitten by *N. kaouthia* with minimal signs of either local or systemic envenoming. One patient, for example, displayed a partial ptosis for 24 hours and a small blister developed at the bite site 5 days after the initial incident. He was not treated with antivenom and did not require respiratory support. He had a concentration of circulating venom antigen of 17.6 ng/mL. Other patients with similarly minimal signs of neurotoxicity had circulating concentrations of venom antigen ranging between 6.1 and 24.3 ng/mL (mean 13.4 ± 3.6). In those patients with clinical signs of severe neurotoxicity, venom antigenemia ranged between 3.5 and 496.5 ng/mL (mean 103.2 ± 16.6; *N* = 39). It seems clear that the severity of neurotoxicity is at least partially dependent on the amount of venom antigen in the circulation. It was not possible to assess whether there was any relationship between the concentration of circulating venom antigen and the speed of onset of neurotoxic signs because patients were usually admitted many hours after the onset of their first sign of neurotoxicity.

It is generally accepted that the clinical syndrome of envenoming by cobras does not include coagulopathy, but in this study two of the 70 patients had incoagulable blood, based on a 20WBCT. However, our detailed study of the possible effects of *N. kaouthia* venom on human blood coagulation using TEG^®^ demonstrated no effect on blood coagulation at venom concentrations exceeding the highest concentration of venom recorded in any patient bitten by a monocled cobra (3,300 ng/mL).^23^ We conclude that the two aberrant 20WBCT results were probably the result of a technical error.

The apparent anemia in most patients was not a consequence of the bite: Bangladeshi people are generally anemic because of a diet short of essential micronutrients.^35^

The use of ligatures and incisions in the emergency treatment of snakebite is universally condemned as being without value and potentially harmful.^19^,^36^–^40^ Bangladeshi clinicians have found it difficult to improve the situation. The positive response to the use of edrophonium in all patients tested suggests that the administration of the long-acting anticholinesterase neostigmine could be recommended as a first aid treatment. More complete training of rural medical and complementary staff would also enable local medical centers to provide assisted ventilation to severely envenomed patients, including oxygen administration and the insertion of an airway (endotracheal, laryngeal mask, or i-gel supraglottic airway). Manual respiratory support has been found to be beneficial in the management of cobra bite.^2^ During the period covered by this present study, 27 patients died en route to CMCH (M. A. Faiz, unpublished data). The problem of unnecessary fatalities is partly due to the difficulty of providing high-quality emergency treatment in rural communities but also to a lack of basic infrastructure and funds to pay for the transport of severely ill patients to a major referral center. Once in CMCH, patient mortality was relatively low (2/70) and similar to case fatalities in similarly specialized facilities elsewhere in southeast Asia.^41^ The problem is common across southeast Asia and a partial solution might be the adoption of motor-cycle ambulances and similar modes of transport that are being developed elsewhere.^21^,^42^–^44^

## Figures and Tables

**Table 1:** Neurological signs of envenoming by *Naja kaouthia* in 70 patients in southeastern Bangladesh

Neurological signs of envenoming		
Sign	Frequency (%)	Duration (hours) mean (maximum)
Ptosis	83	5 (50)
Dysarthria	75	4.5 (30)
Weak neck	64	5 (45)
Difficulty swallowing	59	4 (20)
Blurred vision	42	5 (30)
Double vision	30	6 (35)
Breathlessness	30	7 (50)
Cyanosis	4	n/a

**Table 2:** Average hematological data from 70 patients bitten by *Naja kaouthia* in southeastern Bangladesh compared with the Bangladeshi national reference range

Hematological data			
Cell type		Reference range	Patients
Total white cells		4–11 × 10^9^/L	7–10 × 10^9^/L
Monocytes		2–5%	1–9%
Basophils		0–1%	0%
Eosinophils		1–6%	0–10%
Polymorphs		20–40%	23–90%
Platelets		150–400 × 10^9/^/L	170–380 × 10^9^/L
Hemoglobin	Female	12–16 g/dL	7.5–13.7 g/dL
	Male	14–18 g/dL	5–14.5 g/dL

**Table 3:** Responses to Tensilon^®^ in 18 patients bitten by *Naja kaouthia* in southeastern Bangladesh

Time (minutes)	Ptosis (% iris exposed)		Eye lid retraction (duration: seconds)		Inter-dental cleft (cm)		Peak expiratory flow rate (L/minute)		Blow pressure (mmHg)	
	Control	Tensilon	Control	Tensilon	Control	Tensilon	Control	Tensilon	Control	Tensilon
0	34 ± 5.5	41 ± 5.7	9 ± 1.7	18 ± 3.6*	3.9 ± 0.19	4.1 ± 0.16	49 ± 19	58 ± 17	5.9 ± 2.4	7 ± 2.4
5	33 ± 5.4	49 ± 6.5*	11 ± 1.9	22 ± 5.2*	3.8 ± 0.18	4.1 ± 0.15*	60 ± 19	67 ± 20	6.4 ± 2.4	8.7 ± 3.2*
10	31 ± 5.6	50 ± 6.7*	9 ± 1.6	17 ± 2.0*	3.9 ± 0.18	4.1 ± 0.15	47 ± 20	70 ± 15*	6.6 ± 2.4	8.1 ± 3.1
15	30 ± 5.8	45 ± 7.0*	10 ± 1.8	14 ± 2.3	3.8 ± 0.19	4.0 ± 0.18	54 ± 19	66 ± 20	6.1 ± 2.6	7.8 ± 2.3
20	28 ± 5.1	35 ± 6.1	8 ± 1.5	10 ± 1.8	3.8 ± 0.13	3.8 ± 0.19	44 ± 16	55 ± 20	5.8 ± 2.3	6.8 ± 2.6

All patients were pretreated with atropine sulfate (0.6 mg i.v.). Either Tensilon^®^ (10 mg i.v.) or placebo (normal saline) was given immediately after pretreatment by intravenous infusion over 2 minutes. Thirty minutes later patients received, in reverse order, either Tensilon^®^ or placebo as required.

* The response following the administration of Tensilon^®^ was significantly different from the saline control.
